# Strategies for vitamin B6 biofortification of plants: a dual role as a micronutrient and a stress protectant

**DOI:** 10.3389/fpls.2013.00143

**Published:** 2013-05-21

**Authors:** Hervé Vanderschuren, Svetlana Boycheva, Kuan-Te Li, Nicolas Szydlowski, Wilhelm Gruissem, Teresa B. Fitzpatrick

**Affiliations:** ^1^Department of Biology, Plant Biotechnology, Eidgenössische Technische Hochschule ZurichZurich, Switzerland; ^2^Department of Botany and Plant Biology, University of GenevaGeneva, Switzerland

**Keywords:** vitamin B6, oxidative stress, biofortification, genetic engineering, *PDX* genes, crop biotechnology

## Abstract

Vitamin B6 has an essential role in cells as a cofactor for several metabolic enzymes. It has also been shown to function as a potent antioxidant molecule. The recent elucidation of the vitamin B6 biosynthesis pathways in plants provides opportunities for characterizing their importance during developmental processes and exposure to stress. Humans and animals must acquire vitamin B6 with their diet, with plants being a major source, because they cannot biosynthesize it *de novo*. However, the abundance of the vitamin in the edible portions of the most commonly consumed plants is not sufficient to meet daily requirements. Genetic engineering has proven successful in increasing the vitamin B6 content in the model plant *Arabidopsis*. The added benefits associated with the enhanced vitamin B6 content, such as higher biomass and resistance to abiotic stress, suggest that increasing this essential micronutrient could be a valuable option to improve the nutritional quality and stress tolerance of crop plants. This review summarizes current achievements in vitamin B6 biofortification and considers strategies for increasing vitamin B6 levels in crop plants for human health and nutrition.

## INTRODUCTION

**FIGURE 1:**
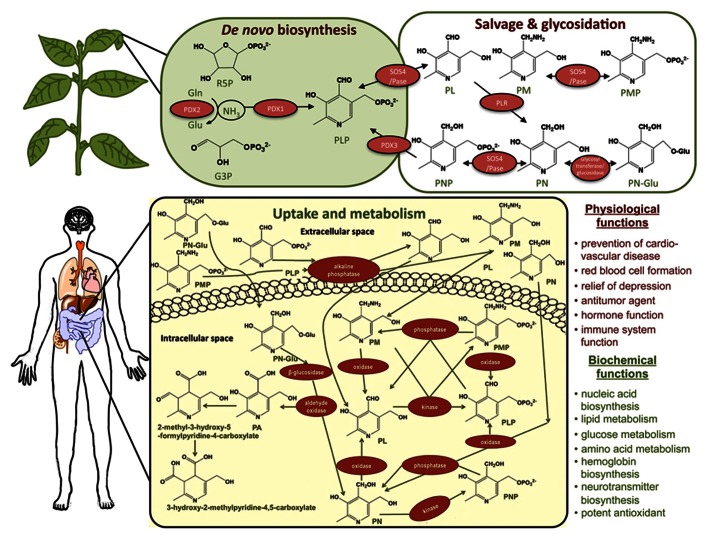
**Plants as a source of vitamin B6 and its uptake by humans**. Plants can biosynthesize vitamin B6 *de novo. *It is predominantly produced in the metabolically active parts of the plant like the leaves but can be found in all organs. The* de novo *pathway (upper left corner) is operating in the cytosol involving only two enzymes: a synthase, PDX1, and a glutaminase, PDX2. The abbreviations used are Glu (glutamate), Gln (glutamine), G3P (glyceraldehyde 3-phosphate), R5P (ribose 5-phosphate), PA (pyridoxic acid), PN (pyridoxine), PN-Glu (PN-glycoside), PL (pyridoxal), PM (pyridoxamine), PNP (pyridoxine 5^′^-phosphate), PLP (pyridoxal 5^′^-phosphate), PMP (pyridoxamine 5^′^-phosphate). The cofactor form of the vitamin, PLP, is produced directly from R5P, G3P, and Gln. A salvage pathway also operates in plants (upper right corner) through which the vitamers can be interconverted. PDX3 is a PNP/PMP oxidase (only the reaction for PNP is depicted), SOS4 is a PN/PL/PM kinase and PLR is a PL reductase. Plants can also convert PN to PN-Glu, a form that is only partially bioavailable to humans, using a glycosyl transferase. In the human body, vitamin B6 is taken up in the jejunum (the second part of the small intestine; lower panel) where the free forms PN, PL, and PM can be taken up directly. The phosphorylated vitamers are dephosphorylated prior to uptake by a membrane bound alkaline phosphatase. In both cases uptake is driven by diffusion, which necessitates the phosphorylation of the vitamers immediately after their internalization to keep a low concentration of the non-phosphorylated forms inside the cells. The PN-Glu is taken up in its glycosylated form and processed by a cytosolic β-glucosidase to release PN. Its low bioavailability is not determined by the uptake itself but by the limiting activity of a β-glucosidase. The uptake is followed by further conversion inside the cells (salvage pathway) yielding the active cofactor form PLP involved in numerous biochemical and physiological processes in the human body (right lower panel). The catabolism of the vitamin involves conversion to PA (as well as more oxidized derivatives as depicted). The latter are excreted in the urine.

The term vitamin B6 refers to a group of six water soluble vitamers (**Figure 1**), namely pyridoxal (PL), pyridoxine (PN), pyridoxamine (PM), and their phosphorylated derivatives. Pyridoxal-5^′^-phosphate (PLP) is the vitamer of central importance because it is required as a cofactor for over 140 chemical reactions in the cell (Hellmann and Mooney, 2010). In particular, it is involved in amino acid, sugar, and fatty acid metabolism (Percudani and Peracchi, 2003). Only plants, fungi, and bacteria can biosynthesize vitamin B6 *de novo*, therefore animals and humans must obtain it from their diet. Various factors such as inadequate food intake, limited dietary diversity, or diseases can cause vitamin B6 deficiency (Di Salvo et al., 2012). Based on the ability of plants to biosynthesize and accumulate vitamin B6 (Chen and Xiong, 2009; Raschke et al., 2011), increasing levels of the vitamin in crops can be a direct way to provide enriched food to the population and reduce the corresponding deficiencies (Martin et al., 2011; Fitzpatrick et al., 2012; Bhullar and Gruissem, 2013). During the last decade, the function and regulation of key genes involved in vitamin B6 biosynthesis have been characterized in the model plant *Arabidopsis*, revealing the importance of the vitamin in plant development, photosynthesis and responses to stress (reviewed in Mooney and Hellmann, 2010). Therefore, the current molecular understanding of vitamin B6 metabolism provides the opportunity of developing crop plants with an increased content of this vitamin beneficial for both human health and improved agronomic performance.

## VITAMIN B6: BENEFITS FOR HUMANS

A vitamin is defined as an organic compound required in limited amounts by an organism that cannot produce it and thus needs to take it up with the diet. The importance of vitamin B6 for human health has been widely reported. Studies have shown that vitamin B6 intake reduces the incidence of important human diseases such as cardiovascular disease, hypertension, epilepsy, diabetes, kidney disease, neurological disorders, and pellagra (Hellmann and Mooney, 2010; **Figure 1**). Furthermore, the cofactor form of vitamin B6 (PLP) may have positive effects on various forms of cancer by strengthening the immune system and delaying tumor progression (Galluzzi et al., 2013). Other studies investigating the impact of vitamin B6 deficiency on health, using the concentration of PLP in the blood as a biomarker, suggest that a low plasma level correlates with multifactorial neurological pathologies such as depression, Alzheimer’s disease, autism, schizophrenia, epilepsy, and Parkinson’s disease (Di Salvo et al., 2012). On the other hand, the beneficial impact of the vitamin on human health is illustrated by reduced mortality and improved health of the elderly with an adequate PLP plasma concentration (O’Leary et al., 2011; Huang et al., 2012). More recently, vitamin B6 deficiency was suggested as the cause of “nodding syndrome,” a disease affecting a growing number of children in Uganda and Southern Sudan (Wadman, 2011; Vogel, 2012). Importantly, vitamin B6 is also a potent antioxidant (Ehrenshaft et al., 1999; Osmani et al., 1999; Bilski et al., 2000). Several studies focusing on the effect of PN on cell cultures indicate that vitamin B6 functions as a protectant against reactive oxygen species produced in the human body (Jain and Lim, 2001; Kannan and Jain, 2004; Mahfouz et al., 2009). Among other factors, malnutrition, pregnancy, diabetes, HIV, alcoholism, and the use of oral contraceptives increase the risk of vitamin B6 deficiency (Merrill and Henderson, 1987; Di Salvo et al., 2012; Fitzpatrick et al., 2012). Limited dietary diversity can accentuate micronutrient deficiencies (Acham et al., 2012) and is the major cause of a vitamin B6 deficit for inhabitants of developing countries. However, inadequate vitamin B6 status has recently been reported in the U.S.A. as well (Morris et al., 2008).

## MAIN SOURCES

Vitamin B6 is present in many food products including meat and vegetables. The major sources of vitamin B6 in Western diets are cereals, poultry, beef, and potatoes (O’Neil et al., 2012). Among the five most important staple crops, potatoes have by far the highest vitamin B6 content (Fitzpatrick et al., 2012). The content is much lower in other staple crops and should be increased several fold (e.g., wheat 12.2-, rice 3.2-, maize 3.5-, and cassava 2.3-fold, respectively) in order to reach the recommended daily allowance (RDA) of vitamin B6 (Fitzpatrick et al., 2012). The bioavailability of vitamin B6 derived from plant food sources is also an important factor influencing adequate intake (**Figure 1**). Plants contain multiple B6 vitamers and a substantial fraction of vitamin B6 is present as pyridoxine-5^′^-β-D-glucoside (PN-glucoside; Gregory and Ink, 1987; Ollilainen, 1999; **Figure 1**). While PN, PL, and PM and their phosphorylated derivatives are fully bioavailable from plant food, PN-glucoside is only 50% bioavailable (Gregory, 2012; **Figure 1**). On the other hand, PN and its glycosylated form are more stable than PL and PLP (the main forms of the vitamin available in animal tissues; Mehansho et al., 1979) and are less susceptible to thermal degradation.

## VITAMIN B6: BENEFITS FOR PLANTS

Unlike animals, plants can biosynthesize their own vitamins with the exception of vitamin B12. In the plant context, therefore, the term vitamin does not correspond to its definition but has been widely used. In plants, as in animals, vitamin B6 is required as a cofactor. There are an estimated 177 PLP-dependent enzymes in *Arabidopsis *(Percudani and Peracchi, 2009; Mooney and Hellmann, 2010). These enzymes have a central role because they participate in universal (e.g., biosynthesis and catabolism of amino acids) as well as plant specific pathways (e.g., starch metabolism, glucosinolate biosynthesis, ethylene and auxin biosynthesis and degradation; Mooney and Hellmann, 2010). To date, the importance of the B6 vitamers *in planta *has mostly been demonstrated from the analysis of mutant plants deficient in vitamin B6 biosynthesis. For example, an *Arabidopsis* mutant, *rsr4-1*, with reduced *de novo* biosynthesis displays altered levels of metabolites such as amino acids and organic acids (Wagner et al., 2006). Moreover, *Arabidopsis *mutants that accumulate lower vitamin B6 levels, i.e., *rsr4-1, pdx1.1, pdx1.3*, are phenotypically distinct from wild-type plants because of impaired seed and seedling development, delayed flowering and reduced plant growth (Chen and Xiong, 2005; Tambasco-Studart et al., 2005, 2007; Titiz et al., 2006; Wagner et al., 2006). Complete biosynthesis knockout mutants (i.e., *pdx2, pdx1.1, pdx1.3*) are embryo lethal (Tambasco-Studart et al., 2005; Titiz et al., 2006). The increased sensitivity of the mutants with reduced levels of vitamin B6 to high concentrations of sucrose, salt, and mannose, as well as high light, UV-B and oxidative stress (Chen and Xiong, 2005; Titiz et al., 2006; Havaux et al., 2009; Ristilä et al., 2011) indicates that vitamin B6 has an important function in plant stress responses as well. Indeed, *Arabidopsis* plants accumulate vitamin B6 when exposed to UV-B (Ristilä et al., 2011) and increased levels of the vitamin in transgenic *Arabidopsis* confer tolerance to oxidative stress (Raschke et al., 2011). The potent *in vitro* antioxidant activities of some B6 vitamers also corroborate this particular function of the vitamin (Ehrenshaft et al., 1999; Bilski et al., 2000; Denslow et al., 2005). In addition, metabolite profiling has revealed increased lipid peroxidation in the *Arabidopsis rsr4-1* mutant deficient in vitamin B6 biosynthesis *de novo *(Lytovchenko et al., 2009), consistent with its role as an antioxidant. Furthermore, the hypersensitive response triggered in tobacco upon incompatible bacterial infection is delayed following leaf infiltration with PN (Denslow et al., 2005). Supplementation of *Arabidopsis *growth medium with the same vitamer reduces singlet oxygen-mediated cell death in the conditional *flu* mutant (Danon et al., 2005). Notably, the phenotype of the latter mutant was also shown to be attenuated in plants with enhanced levels of vitamin B6 (Raschke et al., 2011). Similarly, PN supplementation was demonstrated to restore accumulation of the D1 protein in the *pdx1.3* mutant of *Arabidopsis *(Titiz et al., 2006). As the degradation of this protein is known to be triggered by singlet oxygen generated in the reaction center of photosystem II, its steady state level reflects the oxidative state of the chloroplast. Application of exogenous vitamin B6 on leaf disks also reduces singlet oxygen accumulation caused by high light exposure in wild-type and *pdx1.3* mutant plants (Havaux et al., 2009).

Although vitamin B6 in plants is biosynthesized *de novo* (**Figure 1**), the supplementation experiments discussed above and a similar study using PL, PM, and PN (Huang et al., 2011) suggest that plants can take up exogenously supplied non-phosphorylated B6 vitamers. This is corroborated by the rescue of the root and leaf developmental phenotypes associated with the *Arabidopsis pdx1.1* and *pdx1.3* mutants (Titiz et al., 2006; Wagner et al., 2006) and rescue of the arrest of embryo development at the globular stage in the *pdx2* mutant of *Arabidopsis* by direct application of PN (Tambasco-Studart et al., 2007). The latter study indicates that some transfer of the vitamin occurs between the embryo and the maternal tissue. Very recently a purine permease (PUP1) has been shown to function in recycling of vitamin B6 during guttation (Szydlowski et al., 2013) but no other transporters for this vitamin have been described so far in plants. Since *de novo* vitamin B6 biosynthesis occurs in the cytosol (Tambasco-Studart et al., 2005), diffusion or active transport of vitamin B6 across organelle envelopes is required to support the activity of organellar enzymes dependent on the vitamin as a cofactor (Gerdes et al., 2012). The relatively high polarity of the phosphorylated derivatives of the vitamin does not favor a passive diffusion mechanism (Mooney and Hellmann, 2010) and would require the existence of transporters.

## GENETIC ENGINEERING STRATEGIES TO INCREASE VITAMIN B6 IN CROPS

From a genetic engineering perspective, it is attractive that *de novo* vitamin B6 biosynthesis in plants involves only two enzymes, PDX1 and PDX2 (Ehrenshaft et al., 1999; Ehrenshaft and Daub, 2001; Tambasco-Studart et al., 2005; **Figure 1**). This pathway is also referred to as deoxyxylulose 5-phosphate (DXP)-independent (Tambasco-Studart et al., 2005) to distinguish it from the seven enzyme DXP-dependent pathway first unraveled in *E. coli *(reviewed in Fitzpatrick et al., 2007) and only found in a small subset of bacteria (Ehrenshaft et al., 1999; Mittenhuber, 2001). Interestingly, *Arabidopsis* has three PDX1 homologs, only two of which catalyze vitamin B6 biosynthesis PDX1.1 and PDX1.3 with the function of the third homolog PDX1.2 remaining to be unraveled, and only one PDX2 homolog (Tambasco-Studart et al., 2005). Analyses of the rice and cassava genomes (Ouyang et al., 2007; Prochnik et al., 2012) have revealed an identical number of homologs for both genes.

**FIGURE 2:**
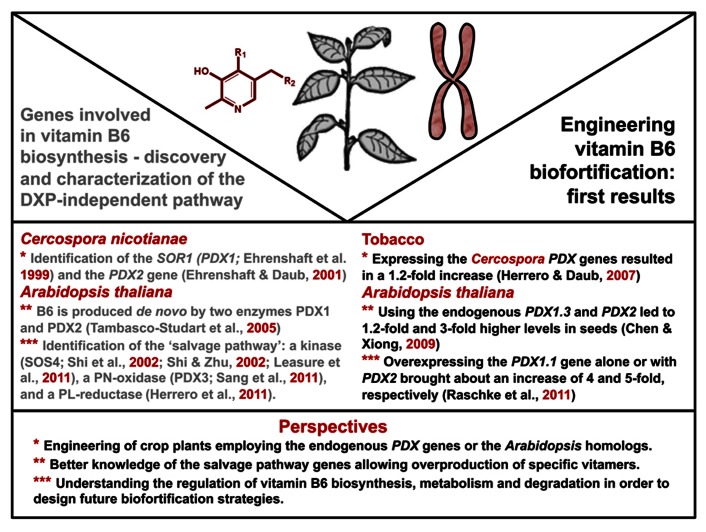
**Summary of the genetic approaches used in unraveling the deoxyxylulose 5-phosphate (DXP)-independent pathway for vitamin B6 biosynthesis *de novo *and first proof-of-principle strategies for vitamin B6 biofortification in *Arabidopsis thaliana* and tobacco**. The lower panel provides an overview of the possible new engineering strategies for vitamin B6 biofortification.

Efforts to increase vitamin B6 accumulation in plants have so far been focused on genes from the DXP-independent pathway. In a first attempt, the *PDX1* and *PDX2* genes from *Cercospora nicotianae *were heterologously expressed in tobacco resulting in a 1.2-fold increase in total vitamin B6 content (**Figure 2**), with some of the transgenic lines displaying delayed seed germination and plant growth (Herrero and Daub, 2007). Moreover, the expression of the endogenous *PDX* genes was altered in these lines, and a tight regulation of vitamin B6 homeostasis in plants was suggested. In another approach, *PDX1.3* and/or *PDX2 *were expressed in *Arabidopsis *under the control of the constitutive 35S cauliflower mosaic virus (CaMV) promoter (Chen and Xiong, 2009; **Figure 2**). A significant increase in total vitamin B6 content was observed only in seeds (1.2-fold) and the transgenic lines overexpressing *PDX2* alone apparently had a higher total vitamin B6 content than the transgenic *PDX1* lines. In this study, gene expression was only assessed at the transcript level. However, the protein level also needs to be analyzed to better understand the mechanism behind these observations. On the other hand, the employment of a seed-specific promoter for the expression of both *PDX1.3* and *PDX2 *resulted in a threefold increase of total vitamin B6 in transgenic seeds (Chen and Xiong, 2009; **Figure 2**). In contrast to the work from Herrero and Daub performed with *Cercospora* genes in tobacco, the latter study reported that ectopic expression of the endogenous *PDX* genes in *Arabidopsis* did not affect plant growth and development. However, the choice of *PDX1 *gene appears to be particularly important considering a more recent report, which describes an at least fourfold increase in total vitamin B6 content in shoots and seeds, respectively using the 35S CaMV promoter (Raschke et al., 2011; **Figure 2**). In this study, it was shown that plants overexpressing *PDX1.1 *at the transcript level also had an increase of the corresponding protein, while *PDX1.3 *transcript overexpression did not result in an increase at the protein level, suggesting a tighter regulation of this paralog. Indeed, PDX1.3 is known to be an ubiquitination target for protein degradation by the proteasome (Manzano et al., 2008). Interestingly, the increase in PDX1.1 protein content was associated with an increased expression of PDX2 and accordingly the vitamin B6 level was increased (Raschke et al., 2011). However, in contrast to the study of Chen and Xiong (2009), overexpression of PDX2 alone did not lead to enhanced levels of vitamin B6 (Raschke et al., 2011). Overexpression of both *PDX1.1* and *PDX2* in *Arabidopsis* led to even further increases in the vitamin B6 content and therefore, appears to be the best strategy for vitamin B6 enrichment in crop plants. Intriguingly, vitamin hyper-accumulator lines also have larger aerial organs as well as increased seed size through embryo enlargement (Raschke et al., 2011). Consistent with the previously reported antioxidant properties of vitamin B6, its accumulation in *Arabidopsis *also correlated with improved tolerance to oxidative stress (Raschke et al., 2011). It is important to mention that the enzymatic activities of PDX1 and PDX2 lead directly to the production of the cofactor form, PLP (Raschle et al., 2005; Tambasco-Studart et al., 2005). However, the enhanced vitamin B6 content in the aforementioned *Arabidopsis* lines was distributed across PN, PM as well as PLP (Raschke et al., 2011). In this context, it should be noted that in addition to *de novo *biosynthesis, plants also have a so called “salvage pathway” that interconverts the different B6 vitamers. The salvage pathway is common to all living organisms (Tanaka et al., 2005; **Figure 1**). In plants, enzymes that are known to be involved include the oxidase PDX3, which transforms PNP or PMP to PLP (Sang et al., 2011); a kinase SOS4, which can phosphorylate PL, PN, or PM (Shi and Zhu, 2002; Shi et al., 2002; Leasure et al., 2011); a PL reductase (Herrero et al., 2011) and as yet unknown, perhaps unspecific phosphatases, which can dephosphorylate the phosphorylated vitamers (**Figure 1**). Therefore, it is likely that there is cross-talk between *de novo* and salvage pathways of biosynthesis, which should be taken into account in any biofortification strategy. Notwithstanding, the vitamin increase reported by Raschke et al. (2011) would be sufficient to meet the RDA in most staple crops (Fitzpatrick et al., 2012). Furthermore, the reduced bioavailability of glycosylated forms must be taken into account in genetic engineering strategies. Future work should also determine whether the fractions of free, phosphorylated and glycosylated B6 derivatives can be modulated. Such approaches will be instrumental in determining whether particular vitamers are associated with improved agronomic traits.

In order to exploit the benefits of an increased vitamin B6 content, the successful strategy used in *Arabidopsis* remains to be demonstrated in a crop plant. This also requires a good understanding of the regulatory mechanisms that control genes involved in vitamin B6 biosynthesis and metabolism, regardless of whether a biofortification strategy employs heterologous or homologous expression. A better knowledge of the natural diversity of vitamin B6 content and availability in crop species could also be instrumental in selecting the *PDX *orthologs providing the highest increase. Comprehensive studies of provitamin A biosynthesis have revealed intra-species genetic mutations linked to differential accumulation of vitamin A precursors that can be exploited to increase vitamin content in crops (Bang et al., 2007; Harjes et al., 2008; Welsch et al., 2010; Yan et al., 2010). Very limited data is currently available on the inter- and intra-species diversity of vitamin B6 accumulation. A recent survey of various wheat genotypes cultivated under different conditions has shown low variation in vitamin B6 content (Shewry et al., 2011) but additional genotypes and species should be investigated to assess vitamin B6 accumulation especially in crop plants. Such evaluation should be performed under controlled conditions given the regulation of vitamin B6 biosynthesis and accumulation by various factors (Denslow et al., 2007; Ristilä et al., 2011).

## ALTERNATIVE CROP PLANTS OF POTENTIAL INTEREST

In order to select appropriate food crops for vitamin B6 biofortification strategies, two criteria should ideally be taken into account: (1) the crop has to be widely used and of economic importance; (2) vitamin B6 accumulation in the consumed part of the crop plant should not be constrained by physiological or developmental limitations. Genetic engineering using *Arabidopsis* suggests that a comparable increase of vitamin B6 can be reached in shoots and seeds (i.e., four to fivefold; Raschke et al., 2011). However, it remains to be demonstrated that similar increases can be reached in rice seeds or potato tubers for example. If successful, the combination of the existing nutritional qualities with a higher level of vitamin B6 would provide additional value to already important staple foods. Based on the findings of Raschke et al. (2011) using *Arabidopsis*, an improvement of plant growth and survival could also be expected but remains to be confirmed for other species. In developing countries, much of the population is highly dependent on mainly one staple crop and therefore particularly at risk of inadequate micronutrient intake (Ruel, 2003; Stupak et al., 2006). Nutritional quality improvement, including fortification of vitamin B6, of these crops could help diminish the occurrence of malnutrition and micronutrient deficiency (Welch and Graham, 2004; Stupak et al., 2006). The generation of crops combining enhanced levels of bioavailable B6 vitamers and better generic traits such as tolerance to oxidative stresses would represent a valuable tool to improve nutrition and food security.

## Conflict of Interest Statement

The authors declare that the research was conducted in the absence of any commercial or financial relationships that could be construed as a potential conflict of interest.
